# Glycogen Synthase Kinase-3 Inhibition Sensitizes Pancreatic Cancer Cells to TRAIL-Induced Apoptosis

**DOI:** 10.1371/journal.pone.0041102

**Published:** 2012-07-19

**Authors:** Shadi Mamaghani, Craig D. Simpson, Pinjiang M. Cao, May Cheung, Sue Chow, Bizhan Bandarchi, Aaron D. Schimmer, David W. Hedley

**Affiliations:** 1 Division of Applied Molecular Oncology, University Health Network, Toronto, Ontario, Canada; 2 Department of Laboratory Medicine and Pathobiology, University of Toronto, Toronto, Ontario, Canada; 3 Ontario Cancer Institute, Princess Margaret Hospital, Toronto, Ontario, Canada; 4 Department of Medical Oncology and Hematology, Princess Margaret Hospital, Toronto, Ontario, Canada; The University of Texas MD Anderson Cancer Center, United States of America

## Abstract

Tumor necrosis factor-related apoptosis inducing ligand (TRAIL) induces apoptosis in a variety of cancer cell lines with little or no effect on normal cells. However, its effect is limited as some cancers including pancreatic cancer show de novo resistance to TRAIL induced apoptosis. In this study we report that GSK-3 inhibition using the pharmacologic agent AR-18, enhanced TRAIL sensitivity in a range of pancreatic and prostate cancer cell lines. This sensitization was found to be caspase-dependent, and both pharmacological and genetic knock-down of GSK-3 isoforms resulted in apoptotic features as shown by cleavage of PARP and caspase-3. Elevated levels of reactive oxygen intermediates and disturbance of mitochondrial membrane potential point to a mitochondrial amplification loop for TRAIL-induced apoptosis after GSK-3 inhibition. Consistent with this, overexpression of anti-apoptotic mitochondrial targets such as Bcl-XL, Mcl-1, and Bcl-2 rescued PANC-1 and PPC-1 cells from TRAIL sensitization. However, overexpression of the caspase-8 inhibitor CrmA also inhibited the sensitizing effects of GSK-3 inhibitor, suggesting an additional role for GSK-3 that inhibits death receptor signaling. Acute treatment of mice bearing PANC-1 xenografts with a combination of AR-18 and TRAIL also resulted in a significant increase in apoptosis, as measured by caspase-3 cleavage. Sensitization to TRAIL occurred despite an increase in β-catenin due to GSK-3 inhibition, suggesting that the approach might be effective even in cancers with dysregulated β-catenin. These results suggest that GSK-3 inhibitors might be effectively combined with TRAIL for the treatment of pancreatic cancer.

## Introduction

The extrinsic apoptosis pathway starts with binding of death receptor ligands such as Fas ligand, tumor necrosis factor-α (TNF-α), and tumor necrosis factor-related apoptosis inducing ligand (TRAIL) to death receptors, activating a series of signals that can lead to two separate, yet interrelated, modes of apoptosis induction. In some cases, activation of death receptor-4 (DR-4) and death receptor-5 (DR-5) leads to the recruitment of adaptor proteins to form the death-inducing signalling complex (DISC), and subsequent activation of the initiator caspases-8 or −10 is sufficient to activate effector caspases-3,−6, and −7, and apoptosis results [1]. However, binding of TRAIL to DR-4 or DR-5 and activation of caspases-8 or −10 is frequently insufficient to induce apoptosis, and requires the release of mitochondrial mediators such as cytochrome C to amplify the death inducing signal [2]. This cross talk between extrinsic and intrinsic apoptosis pathways requires caspase-8 mediated cleavage of the Bcl-2 family member Bid [2], [3]. Truncated Bid (tBid) acts as a blocking mechanism for inhibiting the action of anti-apoptotic Bcl-2 proteins such as Bcl-2, Mcl-1, and Bcl-XL, leading to mitochondria-induced cell death [3].

TRAIL can also bind two sets of non-functional decoy receptors, in which case, the apoptosis induction is blocked [1]. The balance between the death inducing receptors and decoy receptors is a major determinant of apoptosis induction in the target cells [4]. In contrast to TNF-α and Fas, TRAIL appears to show greater specificity towards apoptosis induction in tumor cells *in vivo*, with correspondingly less toxicity to the host [1], [4], [5], [6]. Although the exact mechanism of target specificity by TRAIL is not known, the lack of toxicity in normal cells is partly attributed to the lower expression of death receptors (DR-4 and DR-5) and the increased expression of decoy receptors on the surface of the normal cells [4], [7]. In contrast, a variety of cancers show an increased expression of DR-4 and −5 receptors, rendering them susceptible to TRAIL- induced apoptosis [3]. While the tumoricidal effect of TRAIL is promising, it has limited effects and many tumors are resistant to TRAIL [5], [8], [9], [10], [11].

Previous work by our laboratory and others suggest that NF-κB is positively regulated by glycogen synthase kinase-3 (GSK-3) and is involved in chemo-resistance, cancer cell survival, growth, and metastasis [12], [13], [14]. GSK-3 inhibition induced anti-survival effects in pancreatic cancer cells, associated with downregulation of NF-κB activity and reduced expression of anti-apoptotic target genes such as XIAP, BcL-XL, and cyclin D1 [14].

GSK-3 can also protect cells from TNF-α-mediated cytotoxity, indicating that GSK-3 has a role in blocking death receptor mediated apoptosis [15], [16], [17]. Interestingly, the inhibitory effects of GSK-3 have been extended to other death receptors such as TRAIL and Fas [18], [19]. Inhibition of GSK-3 was shown to potentiate TRAIL-induced apoptosis in human hepatoma and prostate cancer cell lines [10], [20]. In the course of experiments described in our previous work, it was noted that GSK-3 inhibition blocked the activation of NF-κB by TNF-α [14]. Since NF-κB activation has been previously suggested to suppress TRAIL-induced apoptosis in pancreatic cancer cells [21], this finding suggested the potential for GSK-3 inhibition to sensitize pancreatic cancer to TRAIL. In this report, we tested whether GSK-3 inhibition had a sensitizing effect on TRAIL-induced apoptosis both *in vitro* using pancreatic and prostate cancer cell lines and *in vivo* using PANC-1 xenografts.

## Materials and Methods

### Cell Lines and Reagents

Pancreatic adenocarcinoma cell lines PANC-1 and BxPC-3 (ATCC, Rockville, MD) were maintained as described previously [14]. PPC-1 prostate cancer cell lines were cultured in RPMI 1640 containing 10% FBS, 100 units/mL penicillin and 100 µg/mL streptomycin, at 37°C and 5% CO2 in air.

Recombinant human Apo2L/TRAIL was a gift from Genentech, South San Francisco CA. The general caspase inhibitor z-VAD-fmk was from Alexis-Enzo Life Sciences, Inc. Plymouth Meeting, PA.

### Stable Transfections

For overexpression studies, pcDNA3.1-Myc constructs containing anti-apoptotic markers: Bcl-2, BcL-XL, CrmA, and MCL-1 containing pcDNA3.1-His tag construct were used (Sidnet, Toronto, Canada).

PANC-1 and PPC-1 cells were transfected using Lipofectamine 2000 (Invitrogen, Carlsbad, CA) with 0.95 µg/well DNA. Transfectants were selected with G418 for 14 days to generate stable clones as described previously [22]. Functionality of the expressed proteins was confirmed using the staurosporine apoptosis induction assay [23].

### Cell Proliferation Assay

The dose-dependent effect of AR-A014418 (AR-18) (custom synthesized, Toronto Research Chemicals Inc., North York, Ontario, Canada), TRAIL (rh-TRAIL, R&D systems, Inc., Minneapolis, MN) and their combination in PANC-1, BxPC-3 and PPC-1 cells and its genetically modified variants was assessed by the sulphorhodamine B (SRB) dye (Invitrogen, Carlsbad, CA) binding assay as previously described [14] in triplicates and repeated six times.

### Flow Cytometry Analysis of Apoptosis

Combined measurement of mitochondrial membrane potential, generation of reactive oxygen intermediates (ROI), and outer membrane integrity by flow cytometry was as previously described [24]. PANC-1, BxPC-3, and PPC-1 cells were treated with AR-18 (25 µM) for 24 h followed by TRAIL (10 ng/mL) for 24 h, with single agent or untreated controls. Cells were stained with (40 nM) DiIC1(5) (Invitrogen, Carlsbad, CA) and dichlorodihydrofluorescin diacetate (H2-DCFDA) (5 µM) (Invitrogen, Carlsbad, CA), and Propidium iodide (PI) at a final concentration of 1 mg/mL.

### Genetic Knockdown of GSK-3

GSK-3 isoforms were genetically knocked down in PANC-1 cells transfected with siRNA against GSK-3 α and β isoforms using a reverse transfection protocol as described previously [14].

### Pancreatic Cancer Xenograft Model

Experiments were done according to regulations of the Canadian Council of Animal Care and institutional guideline for animal welfare. PANC-1 xenografts were established subcutaneously in the flanks of 6-week-old male severe combined immunodeficient (SCID) mice, and allowed to grow to about 10 mm in the largest diameter. Tumor volume was calculated using the formula: length × width^2^ × 0.5 [25]. Mice were randomly assigned to 4 groups (n = 5) to receive AR-18 20 mg/kg (5 mg/mL stock in DMSO) twice daily for two days, followed by a 24 h resting period before i.p. injection of TRAIL 25 mg/kg, or single agent or vehicle control groups. Animals were weighed and assessed for signs of toxicity daily, and sacrificed on day 5. Tumors were excised, pieces snap frozen, formalin fixed or lysed as described below.

### Immunoblotting

Western blotting procedure was performed as described previously [14] using rabbit polyclonal antibodies against cleaved caspase-3, β-catenin, cleaved PARP, Bcl-2, BcL-XL, (Cell Signaling Technology, Danvers, MA), and mouse monoclonal antibody against GSK-3 α/β (Biosource Inc., Camarillo, Canada).

### Immunohistochemistry and Image Capture

Tissues from liver, kidneys and tumor were formalin fixed, paraffin embedded, and processed as 4 µm sections. Serial sections were immunostained using H&E and cleaved caspase-3 as described previously [26] and visualized under an Olympus BX41 microscope using 4X and 40X magnification objective lenses.

### Quantification of Cleaved Caspase-3

Slides stained for cleaved caspase-3 were analyzed in a blinded manner to obtain an H-score. The staining intensity of each specimen was judged relative to the intensity of a control slide from mice receiving vehicle treatment. A score of 1+ indicated weak staining relative to background, 2+  =  moderate staining, and 3+  =  strong staining. Staining intensity was reported at the highest level of intensity observed in all tissue elements. For comparison of staining among tissues, the results were quantified by calculation of a complete H-score that considers both staining intensity and the percentage of cells stained at a specific range of intensities. A complete H-score was calculated by multiplying the intensity into the percentage positively stained cells (H = P×I) as described previously [26].

### Statistical Analysis

To investigate the possible synergistic effect of combining AR18 with TRAIL, the interaction between the two drug treatments was tested by fitting it into a model that considers the fact that some experiments were not performed at the same time. The values of optical density (for SRB) were log transformed to stabilize the variance of the residuals. The resulting values were analysed by comparing between different concentrations of each drug using linear regression models. A drug interaction was considered synergistic when the effect of the drug combination was significantly greater than the sum of the effects of both drugs, and sub-additive when it was less than that. Treatment effects were compared using Student's t test and differences between means were considered to be significant when P≤0.05. The analysis was performed by the help of “R” software as described previously [14]. Results were expressed as mean ± SE.

Statistical analysis of the complete H-scores was performed using the two-tailed Student's t-test with unpaired data of equal variance. Statistical significance were considered where value p = <0.05.

## Results

### GSK-3 Inhibition Sensitizes TRAIL- Resistant Pancreatic Cancer Cells to Apoptosis

**Figure 1:**
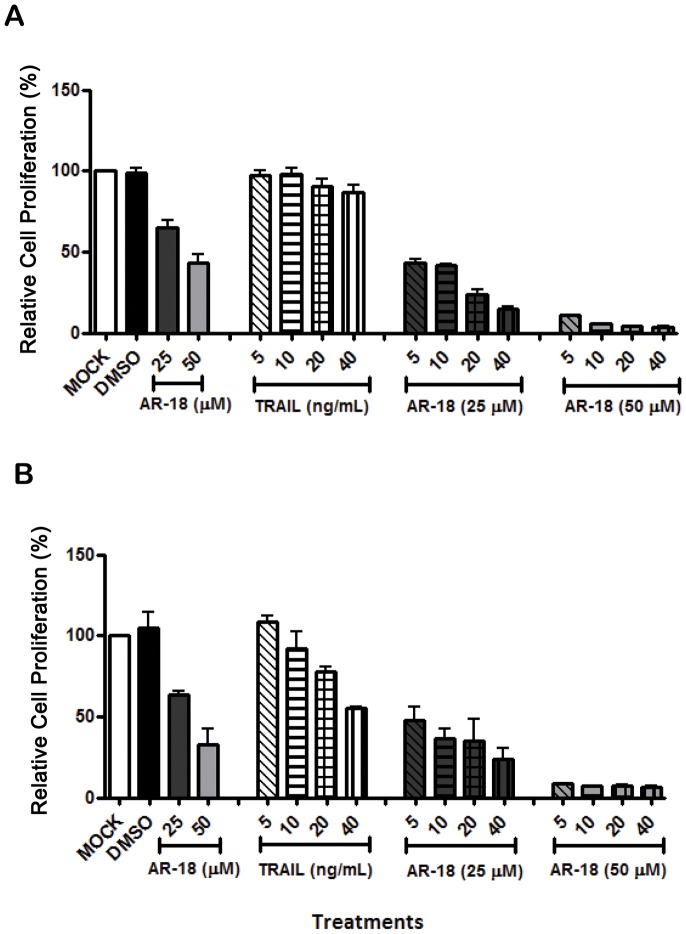
GSK-3 inhibition sensitizes TRAIL-resistant pancreatic cancer cells to apoptosis. PANC-1 (A) and BxPC-3 (B) were treated with TRAIL after 24 h pre-exposure to AR-18 at the indicated concentrations, and cell proliferation measured by SRB assay. Each bar graph signifies mean from three separate experiments with six replicates. error bars  =  ± SEM.

Treatment of PANC-1 and BxPC-3 cells with AR-18 (25 and 50 µM) or DMSO for 24 h, followed by the addition of TRAIL (5, 10, 20, 40 ng/mL) for a further 24 h revealed that treatment with both drugs leads to concentration-dependent growth inhibition as determined by the SRB assay, although PANC-1 cells were relatively resistant to TRAIL (Figure 1). Substantially greater toxicity was seen in both cell lines upon combination of the two agents, and this effect is highly synergistic when analyzed as described previously [14] (for BxPC-3, p = 0.0002 using the combination of 40 ng/mL TRAIL and 50 µM AR-18 and p<0.005 for all the remaining dose schedules; for PANC-1, p<0.005 for all dose schedules except for 25 µM AR-18 combined with 5 and 10 ng/mL TRAIL which was not significant) (Figure 1 and Table S1). The effect was more evident following pre-treatment with 50 µM AR-18, suggesting that the synergistic effect depends on the extent of GSK-3 inhibition rather than TRAIL concentration.

Immunoblot analysis of PARP cleavage in cell lysates extracted from PANC-1 and BxPC-3 cells treated with 25 µM AR-18, 10 ng/mL TRAIL, or the combination showed that while there was no detectable PARP cleavage using AR-18 alone, the combined treatment with TRAIL significantly enhanced PARP cleavage in both cell lines. Consistent with the effects observed by SRB assay, BxPC-3 cells showed sensitivity to TRAIL based on PARP cleavage, whereas PANC-1 cells were resistant.

**Figure 2:**
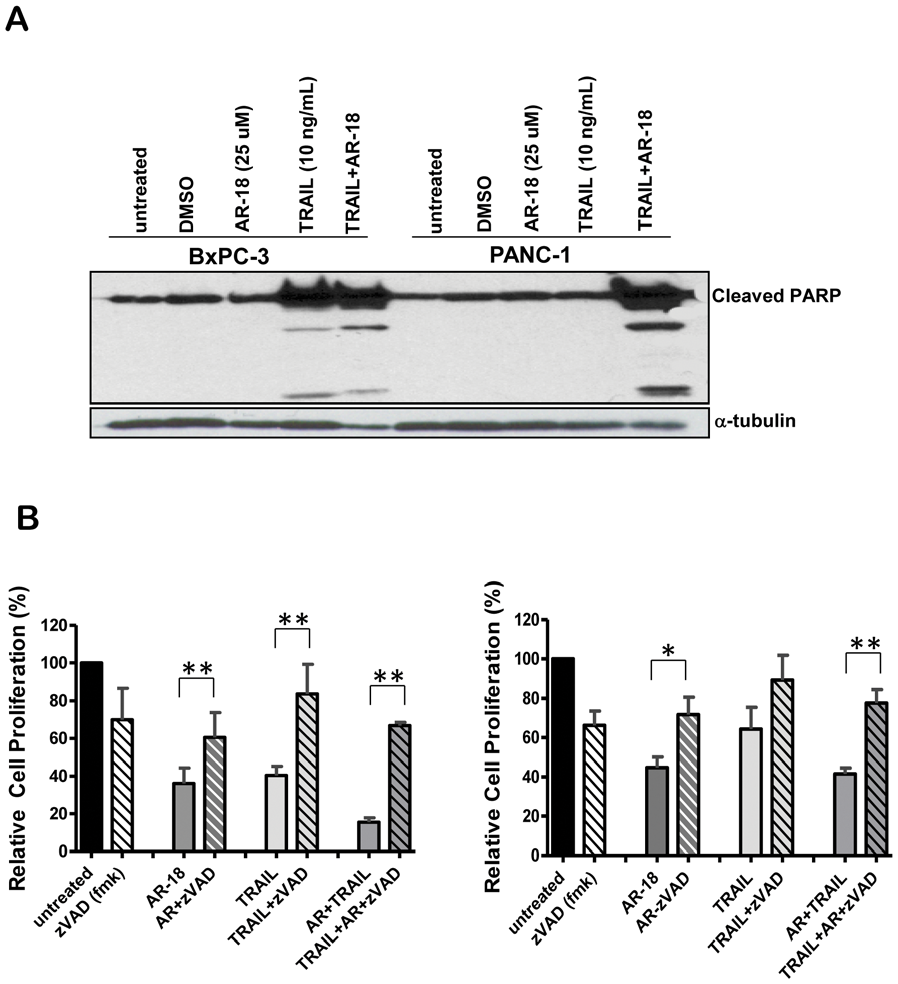
GSK-3 inhibition enhances TRAIL sensitization through PARP and caspase cleavage. (A) BxPC-3 and PANC-1 cells were treated with AR-18, in combination with or without TRAIL for 24 h and analysed by for PARP cleavage. (B) BxPC-3 (left) and PANC-1 (right) cells were treated with AR-18 (25 uM), TRAIL (10 ng/mL), z-VAD-fmk 50 µM, or the combinations indicated and SRB assay after 22 h incubation. Each bar graph signifies mean from three experiments with six replicates. error bars  =  ± SEM. *p<0.005, **p<0.0001.

To test if the TRAIL sensitization of pancreatic cancer cells by GSK-3 inhibition is caspase-dependent, PANC-1 and BxPC-3 cells were treated with 25 µM AR-18 and/or 10 ng/mL TRAIL in the presence of the general caspase inhibitor z-VAD-fmk. As shown in Figure 2B, although z-VAD itself produced some toxic effects, yet it significantly rescued both the cell lines from the AR-18 plus TRAIL combination. Furthermore, TRAIL had a caspase-dependent effect on BxPC-3 but not PANC-1 cells, consistent with their greater TRAIL sensitivity shown by SRB assay. As seen in Figure 2B, partial rescue from AR-18 occurred with z-VAD-fmk, suggesting that the inhibitory effects of GSK3 inhibition are, to some extent, caspase-dependent. These data indicate that the TRAIL sensitization by GSK-3 inhibition involves caspase activation.

### Effects of Genetic Knockdown of GSK-3 on TRAIL Sensitization of Pancreatic Cancer Cells

**Figure 3:**
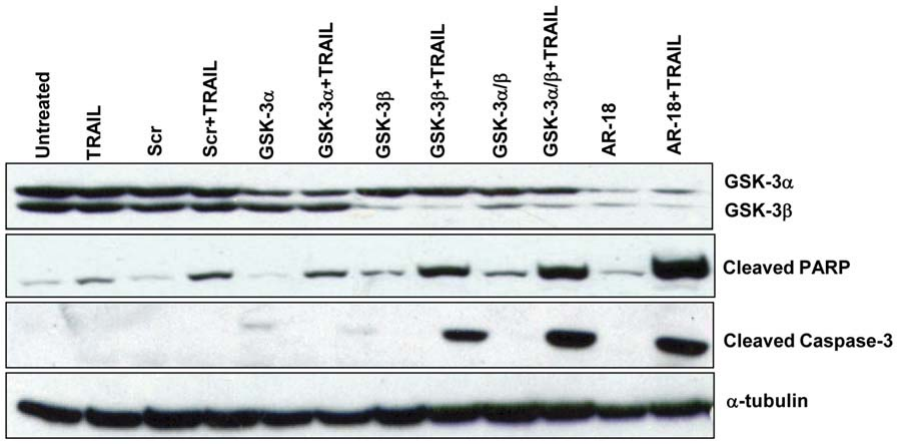
Genetic knockdown of GSK-3 renders cells sensitive to TRAIL-induced apoptosis. PANC-1 cells were pre-treated using AR-18 or siRNA against both isoforms of GSK-3, and then exposed to TRAIL. Immunoblotting confirms successful knockdown of GSK3α and β. This resulted in increased cleavage of PARP and caspase-3 following exposure to TRAIL. Scr: Scrambled non-specific siRNA.

To test if the TRAIL-sensitizing effect of AR-18 was due to GSK-3 inhibition, and to test for functional redundancy in the GSK-3 isoforms, we next examined the expression levels of cleaved PARP and cleaved caspase-3 in cell lysates from PANC-1 cells transiently transfected with siRNA targeted against GSK-3α, β, or both, in the presence or absence of 10 ng/mL TRAIL. Cells transfected with scrambled siRNA or receiving no treatment were used as negative controls, while cells treated with AR-18 alone or in combination with TRAIL were considered the positive control. Immunoblotting showed >80% reduction of total protein of each isoform (Figure 3) in response to 3-day incubation with siRNAs against GSK-3 isoforms if compared to the untreated or scrambled treated cells. Treatment with TRAIL (10 ng/mL) alone had a minor effect on apoptosis as measured by PARP or caspase-3 cleavage, whereas transient knockdown of GSK-3 α or β isoforms alone had no significant effects. The combination of TRAIL with GSK-3β genetic knockdown, in contrast, substantially enhanced PARP and caspase-3 cleavage when compared to untreated, single treatments, or scrambled siRNA. GSK-3α knockdown in combination with TRAIL induced minor effects on PARP and caspase-3 cleavage, but simultaneous knockdown with GSK-3 had a significant effect that was comparable to that of AR-18 (Figure 3). These results support the idea that GSK-3 inhibition is responsible for the TRAIL sensitizing effects of AR-18, and further suggest that GSK-3β plays a more prominent role. However, the role of GSK-3α is less clear because we were not able to achieve the same degree of knockdown as GSK-3β.

### TRAIL Sensitization upon GSK-3 Inhibition Involves Mitochondria

**Figure 4:**
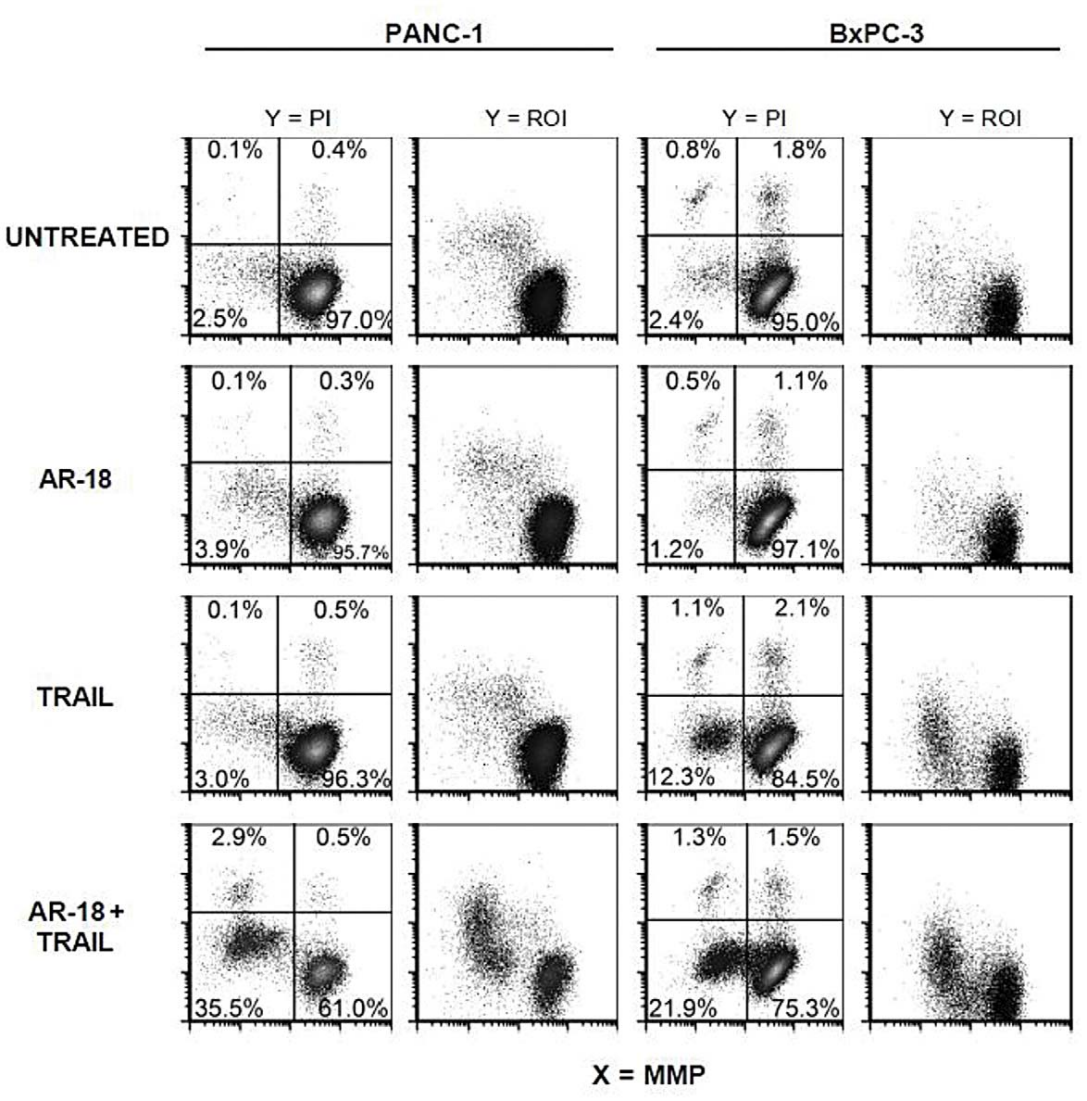
TRAIL sensitization by AR18 is associated with loss of ΔΨm and increased ROI generation. PANC-1 and BxPC-3 cells were untreated or incubated with AR-18 (25 µM), TRAIL (10 ng/mL), or their combination for 24 h, and then analyzed by flow cytometry. Dot density plots of propidium iodide (PI) uptake vs ΔΨm show that loss of ΔΨm precedes PI uptake. TRAIL induced loss of ΔΨm in BxPC-3 but not in PANC-1, consistent with their greater sensitivity seen using the SRB assay (Figure 1). Combined treatment with TRAIL and AR18 clearly sensitized both cell lines to loss of ΔΨm, and this was accompanied by increased ROI generation and loss of out membrane integrity.

Death receptor signalling initiates a cascade of events that results in the activation of the effector caspase-3 and the induction of apoptosis [6]. However, in some cells caspase-3 activation requires signal amplification via Bid cleavage and mitochondria activation [2], [6]. This in turn leads to an imbalanced physiological status of the mitochondria resulting in increased generation of reactive oxygen intermediates and perturbation of the mitochondrial membrane potential [24]. To test for the involvement of the mitochondria during TRAIL sensitization by AR-18, flow cytometry was used to measure mitochondrial membrane potential (ΔΨm) and ROI generation. Combination of TRAIL and AR-18 induced formation of heterogeneous populations of PANC-1 and BxPC-3 cells that had the characteristics of increased ROI generation, loss of ΔΨm, and PI uptake, when compared to untreated control or single treatments (Figure 4). Consistent with results from the SRB assay and PARP cleavage, AR-18 treatment did not elicit apoptotic features in any of the tested cell lines. Treatment with TRAIL alone produced some loss of ΔΨm and increased ROI in BxPC-3 but not PANC-1 cells, although this effect was not as extensive as the combination treatment. These results suggest that TRAIL sensitisation by GSK-3 inhibition involves the mitochondria.

### Molecular Mechanisms of TRAIL Sensitization by GSK-3 Inhibition

**Figure 5:**
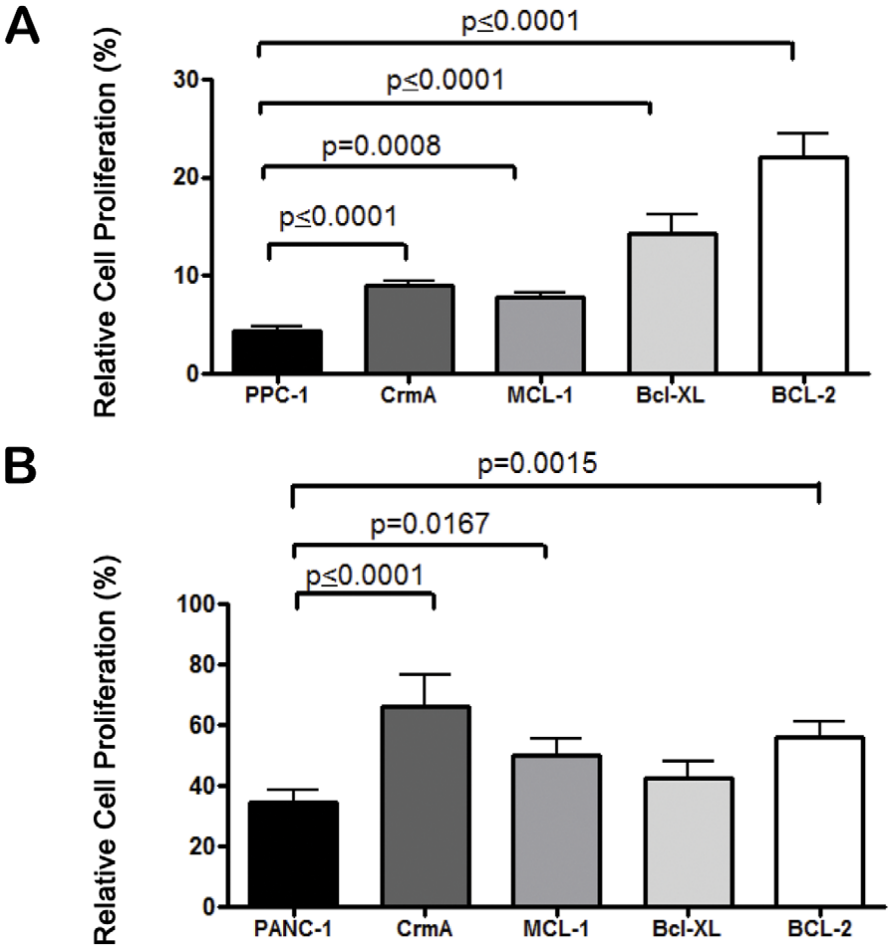
Effects of overexpression of CrmA, MCL-1, Bcl-XL, and BCL-2 on TRAIL sensitization by AR-18. PPC-1 (A) and PANC-1 (B) stable clones transfected with Bcl-2, BcL-XL, CrmA, and MCL-1 containing pcDNA3.1-His tag construct were treated with the combination of AR-18 (50 µM) and TRAIL (10 ng/mL) for 24 h. Results are expressed as cell viability measured by the SRB assay. Each graph signifies mean from three experiments with six replicates. error bars  =  ± SEM.

To further investigate the mechanisms of TRAIL sensitization by GSK-3 inhibition, we employed PPC-1 prostate cancer cell overexpressing Bcl-2, Bcl-XL, and Mcl-1 to inhibit the mitochondrial pathway, or cytokine response modifier A (CrmA) to inhibit the death receptor pathway of caspase activation. Assessment of wild type PPC-1 cells using the SRB and flow cytometry assays showed that the drug combination was more effective than the single agents (Figure S1). Next, we evaluated the effects of the anti-apoptotic proteins on sensitivity to TRAIL and AR-18. PPC-1 cells overexpressing CrmA, Mcl-1, Bcl-XL, and Bcl-2 showed significantly enhanced cell proliferation following treatment with the TRAIL + AR-18 combination, when compared to cells not overexpressing any anti-apoptotic markers (Figure 5A).

Similar to the results obtained using the PPC-1 cells, overexpression of CrmA and Bcl-2 in PANC-1 cells protected from the TRAIL + AR-18 combination, although there were differences in the relative effects of the Bcl-2 family members and the effects of Bcl-XL were not statistically significant in PANC-1 cells. The protective effect overexpressing CrmA, which acts to suppress death receptor caspase activation, suggests the possibility that GSK-3 inhibition might sensitize to TRAIL through enhancement of initiator caspases such as caspase-8, as well as via anti-apoptotic molecules such as Bcl-2 and Mcl-1 in pancreatic cancer cells. However, further work would be needed to establish this.

### AR-18 Sensitizes PANC-1 Cells to TRAIL *in vivo*

**Figure 6:**
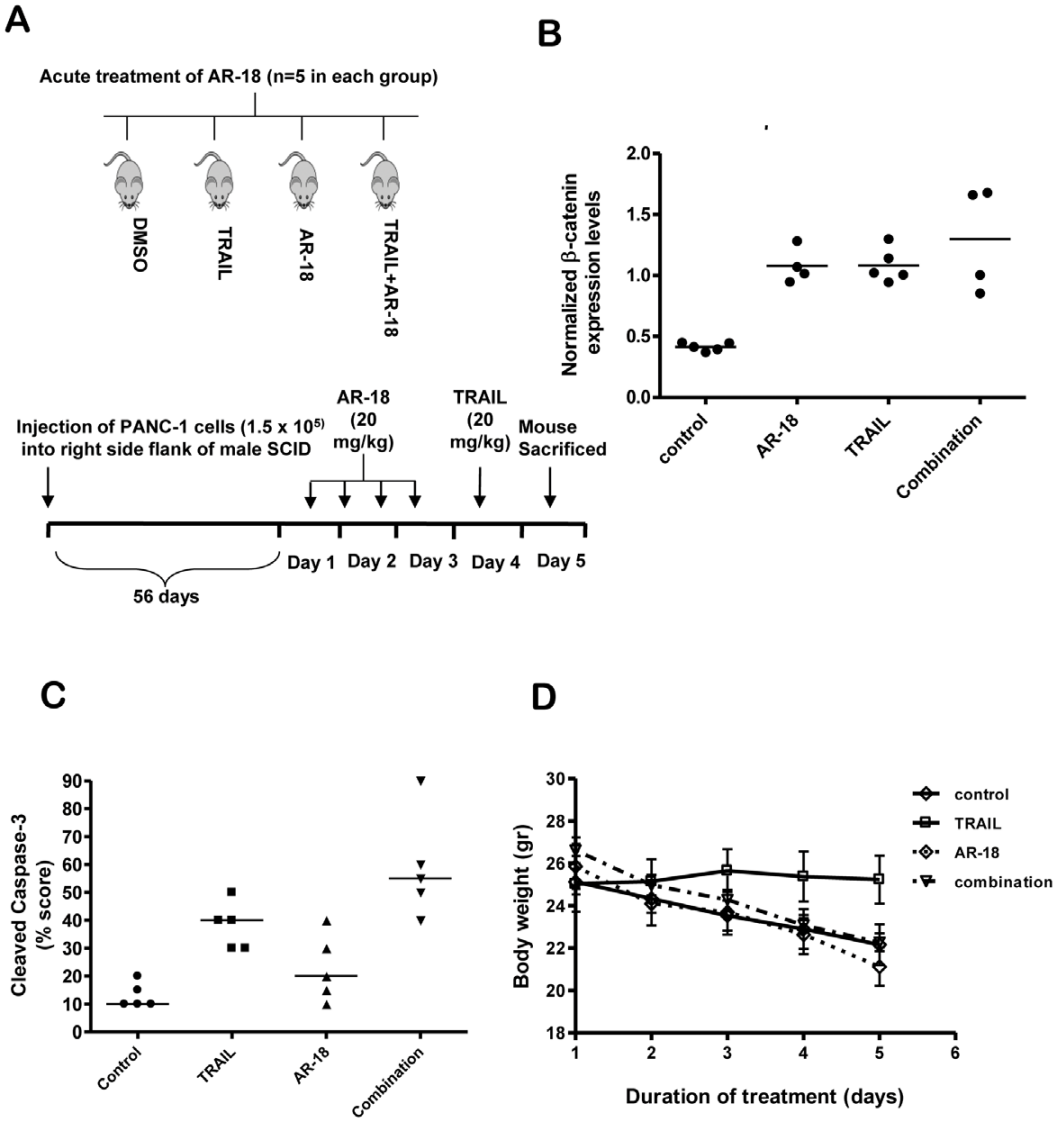
AR-18 sensitizes PANC-1 xenografts to TRAIL. (A) Schematic of *in vivo* treatment protocol. (B) β-catenin expression levels were quantified using densitometry and the resulting values were normalized against α-tubulin. Student’s t-test indicated that combination vs control: p = 0.0012, and for AR-18 vs control: p<0.0001. (C) Quantification of apoptosis in the tumor samples using H- score. Cleaved caspase-3 stained slides (as shown in Figure S2) were scored for intensity of the staining (I) as well as percentage of cells positive for the corresponding stain (H = I×P). Results of unpaired student t-test (n = 5 in each treatment group) are as follows: TRAIL vs control (p = 0.0002), combination vs control (p = 0.0004), combination vs TRAIL (p = 0.0261), combination vs AR-18 (p = 0.0035). (D) Evaluation of animal weight during treatment.

In order to examine the effect of short term exposure to the combination of AR-18 and TRAIL on apoptosis induction in PANC-1 xenograft tumors, we first established that the maximum dose of AR-18 tolerated by SCID mice used by our laboratory was 20 mg/kg, given twice daily by intraperitoneal injection (i.p.). Male SCID mice bearing PANC-1 xenografts were divided into four different treatment groups and treated i.p. injection with DMSO or AR-18 (20 mg/kg; every 12 hours for 2 days) followed by i.p. injection of TRAIL (25 mg/kg) within 24 h after the last AR-18 dose. 24 hours after the last treatment mice were sacrificed and tumors harvested (Figure 6A).

As readout for the pharmacodynamic effects of AR-18, we measured the level of β-catenin which is regulated by GSK-3 through the Wnt pathway. There was a significant (p<0.0001) increase in β-catenin levels when compared to DMSO treated samples following treatment with AR18 (Figure 6B), suggesting that GSK-3 inhibition was successfully achieved in the PANC-1 xenografts. Unexpectedly, the levels of β-catenin were significantly increased in TRAIL treated mice. This appears to be a novel finding, the significance of which is currently uncertain. Furthermore, the combination of both drugs significantly increased β-catenin levels when compared to DMSO controls, but the values were not significantly higher than AR-18 alone.

### Combination Therapy Increases Cleaved Caspase-3 *in vivo*

The H-score measurements of cleaved caspase-3 indicated that TRAIL alone produced significant (p = 0.0002) apoptosis induction in PANC-1 tumors (Figure 6C and Figure S2), whereas AR-18 as a single agent had insignificant effects. However, the combination of AR-18 and TRAIL showed a larger effect in inducing apoptosis when compared to single treatments or DMSO treated controls: combination vs. control (p = 0.0004), combination vs. TRAIL (p = 0.0261), combination vs. AR-18 (p = 0.0035). To investigate any potential toxic effects in the animals receiving 20 mg/kg AR-18 alone or in combination with TRAIL, liver and kidneys were formalin fixed, paraffin embedded and sections were stained with H&E and cleaved caspase-3. No obvious morphological changes of injury including necrosis or apoptosis were seen. The body weight of the animals measured daily over the course of this acute dosing experiment show no significant differences between treatment groups, except that animals receiving TRAIL alone maintained their weigh when compared to DMSO control (Figure 6D).

## Discussion

This study suggests the potential to sensitize pancreatic cancers to TRAIL by GSK-3 inhibition. First, we observed TRAIL sensitization via GSK-3 inhibition in both prostate and pancreatic cancer cell lines. Second, the process of TRAIL sensitization was shown to be caspase-dependent. Third, genetic knockdown of GSK-3 using isoform specific siRNAs was equally effective in TRAIL-sensitization as was the small molecule inhibitor AR-18. Fourth, GSK-3 inhibitor-induced TRAIL sensitization was associated with the loss of mitochondrial membrane potential accompanied by increased ROI generation. Fifth, overexpression studies in pancreatic cancer cells indicated that both caspase-8 (CrmA) and NF-κB-dependent mitochondrial proteins (Bcl-2, and Mcl-1) were involved in the process of TRAIL sensitization. Use of TRAIL in combination with GSK-3 inhibition has relevance to the treatment of pancreatic cancer as these tumors have a high frequency of K-ras and p53 mutations that likely contribute to their resistance to standard agents [27], [28], whereas TRAIL sensitivity is reported to be p53-independent and TRAIL is effective in tumors with inactive p53 [29]. Also, activating K-ras mutations have been reported to sensitize cancer cells to TRAIL-induced apoptosis [30], [31].

We first tested the TRAIL sensitization phenomenon and optimized our study using prostate cancer cells because of the previous proof of concept in these cancers [20]. Interestingly, although there was variation in TRAIL sensitivity among the tested cell lines, with PANC-1 being the most TRAIL resistant, the combination with GSK-3 suppression provided a consistent and highly significant sensitization to TRAIL in all the cell lines tested. This sensitization appeared to be more dependent on the level of GSK-3 inhibition, rather than on TRAIL concentration.

Previous studies attributed the TRAIL resistance of pancreatic cancer cells to the overexpression of X-linked inhibitor of apoptosis (XIAP) [32]. Volger *et al.* showed that blocking XIAP sensitized pancreatic cancer cells to TRAIL-induced apoptosis both *in vitro* and *in vivo* [33]. Other studies have emphasized the role of NF-κB in resistance to TRAIL through the upregulation of inhibitors of apoptosis such as XIAP, Bcl-2, Bcl-XL, and Mcl-1 [21], [34], [35], [36], [37]. GSK-3 inhibition downregulates the expression level of these NF-κB target genes [14], [38] and can also block the death receptor pathway [15], suggesting that GSK-3 inhibition might sensitize cancer cells to TRAIL treatment through multiple mechanisms.

In line with the previous finding that GSK-3 can block death receptor signalling upstream of caspase-8 [15], CrmA overexpression rescued from the TRAIL + AR18 combination. Although suggestive of an additional role for GSK-3 in death receptor signalling, further experiments would be needed to confirm that. We also found cleavage of caspase-3 and PARP in cells sensitized to TRAIL by either AR-18 or genetic knockdown of GSK-3 isoforms. Interestingly, the effect of GSK-3β in TRAIL-induced apoptosis was more prominent than GSK-3α, although the intensity of double isoform knockdown was significantly enhanced when compared to GSK-3β alone, and was comparable to that seen with AR-18. These results were similar to our previous report indicating that genetic blockade of GSK-3β and double knockdown of GSK-3 isoforms are relatively more effective than GSK-3α in suppressing NF-κB activity [14]. However, that was not formally tested in these experiments and would require further investigation.

Several of the NF-κB target proteins inhibit the mitochondrial pathway, and we therefore asked if the AR-18+ TRAIL combination disrupts mitochondrial function. We found an enhanced release of ROI in conjunction with decreased mitochondrial membrane potential in cells treated with TRAIL + GSK-3 inhibitor, suggesting the involvement of mitochondria. Although generation of ROI as an effector mechanism during drug-induced apoptosis in cancer cells is well known [39], our results are of interest as they suggest a link between TRAIL sensitization by GSK-3 inhibition and the mitochondrial respiratory chain. Such an effect was previously observed in a study by Jung *et al*., using curcumin to sensitize renal cancer cells to TRAIL [40]. Curcumin treatment enhanced ROI generation in TRAIL sensitized cells. Additionally, we also observed an increase in DR-5 expression in a ROI-dependent manner [40]. Similar results were previously reported in human astroglial cells, emphasizing ROI-dependent up-regulation of TRAIL death receptors [41]. Cleavage of caspase-3 observed in our study might further influence ROI production, as activated caspase-3 can induce a feedback loop on the mitochondrial respiratory chain [42]. Whether increase in ROI generation in pancreatic cancer cells is the crucial cause of TRAIL sensitization after GSK-3 inhibition, or it is generated as a bystander effect of apoptosis execution, remains to be investigated. It is also worth noting that ROI generation might again link NF-κB to the process of TRAIL sensitization of GSK-3 inhibitors in pancreatic cancer. Because GSK-3 inhibition blocks NF-κB, the ROI generation might be a subsequent effect to NF-κB inhibition, an effect observed using curcumin to sensitize renal cancer cells to TRAIL [40].

Anti-apoptotic Bcl-2 family proteins Mcl-1, Bcl-XL, and Bcl-2 have been reported to be highly expressed in pancreatic cancer cells, and to contribute to apoptosis resistance [9], [43]. Targeted overexpression studies using Bcl-2, Bcl-XL, and Mcl-1 indicate that NF-κB associated mitochondrial anti-apoptotic molecules play a significant role in TRAIL resistance, although there is variation between PANC-1 and PPC-1 cells, and support an anti-apoptotic role of GSK-3 consistent with previous reports by our group and others that GSK-3 inhibition downregulates Bcl-2 anti-apoptotic proteins [14], [38].

Next, we investigated whether GSK-3 inhibition sensitizes tumors to TRAIL *in vivo*. We established a dose of AR-18 that was tolerated during short-term treatment, and confirmed GSK-3 inhibition by measuring the level of β-catenin in tumor tissue [14], [44]. Although short-term treatment with AR-18 was insufficient to induce apoptosis, it sensitized PANC-1 xenograft tumors to TRAIL induced apoptosis as determined by increased caspase 3 cleavage. Unexpectedly, TRAIL significantly increased the expression of β-catenin in the PANC-1 xenografts. β-catenin plays an important role in tumorigenesis [45], [46]. Previous studies have suggested a close connection between increased expression of β-catenin and TRAIL resistance [47]. It is therefore possible that the increased levels of β-catenin in response to TRAIL are part of a response that promotes cellular resistance. However, the combination of AR-18 and TRAIL activated caspase 3 to a greater extent than the single agents, suggesting that the pro-apoptotic effect of the combination outweighs any anti-apoptotic effects of β-catenin induction by TRAIL.

In the present paper we focused on the effects of GSK-3 linked to NF-κB activation to explain the TRAIL-sensitizing effects of GSK-3 inhibition, based on our earlier work and that of others [14], [21]. However, we recognize that GSK-3 has multiple additional roles that might affect cell survival, for example through Wnt signalling, microtubule assembly or cell cycle progression that were not tested in this work [48], [49]. Because of the multi-tasking nature of GSK-3, it is possible that additional or unpredictable toxic effects might occur as a result of drug treatment, and will require further investigation. In our hands, *in vivo* use of AR-18 exhibited severe toxic effects using doses that were much lower than those previously described [50]. At the present time, it is not clear if this results from off-target effects of AR-18, or is the consequence of selective GSK-3 inhibition. The *in vivo* pharmacology of AR-18 is not well documented to date and our limited data using chronic dosing suggests that this agent has a narrow therapeutic window. Therefore, we believe that future investigation of the pre-clinical (and potential early clinical) effects of the combination with TRAIL will require a GSK-3 inhibitor that shows greater selectivity and drug-like properties. With this reservation, however, we believe that the combination has potential as a novel approach for testing in the clinic.

## Supporting Information

Figure S1 **AR-18 sensitizes wild-type prostate cancer cell line PPC-1 to TRAIL.** Left panel: PPC-1cells were treated with TRAIL for 24 h after a 24 h pre-exposure to AR-18 at the indicated concentrations, and cell viability measured by SRB assay. Each bar graph signifies mean from three separate experiments with six replicates. error bars  =  ± SEM. Right panel: PPC-1 cells were untreated or incubated with AR-18 (25 µM), TRAIL (10 ng/mL), or their combination for 24 h, and then analyzed by flow cytometry. TRAIL induced loss of ΔΨm and combined treatment with TRAIL and AR18 clearly sensitized the cell line to loss of ΔΨm, and this was accompanied by increased ROI generation and loss of out membrane integrity.
(TIF)

Figure S2 **Synergistic interaction of GSK-3 and TRAIL in apoptosis induction** ***in vivo*** **.** Immunohistochemistry staining of cleaved caspase-3 and H&E in PANC-1 s.c. tumor xenografts in male SCID mice. Groups of five mice were i.p. treated with acute doses of DMSO (control), AR-18, TRAIL or their combination, as depicted in Figure 6A. Tumors were excited, formalin fixed, stained and visualized under an Olympus BX41 microscope. A representative section imaged using a 40X magnification objective lens from one mouse in each group is presented to the right.
(TIF)

Table S1 **Statistical analysis indicates synergistic interaction when AR-18 and TRAIL are combined.** To determine the potential synergistic effect of the AR-18 and TRAIL combination, the SRB cell proliferation data from PANC-1 and BxPC-3 (Tables A and B respectively) were subjected to statistical analysis by log transforming the data and using linear regression model. Syn: synergistic effect, Non-syn: Not synergistic.
(TIF)
