# Supplementary figures and images for: Glycogen Synthase Kinase-3 Inhibition Sensitizes Pancreatic Cancer Cells to TRAIL-Induced Apoptosis
Source: PLoS One. 2012 Jul 19;7(7):e41102. doi: 10.1371/journal.pone.0041102 (PMC3400624; doi:10.1371/journal.pone.0041102)

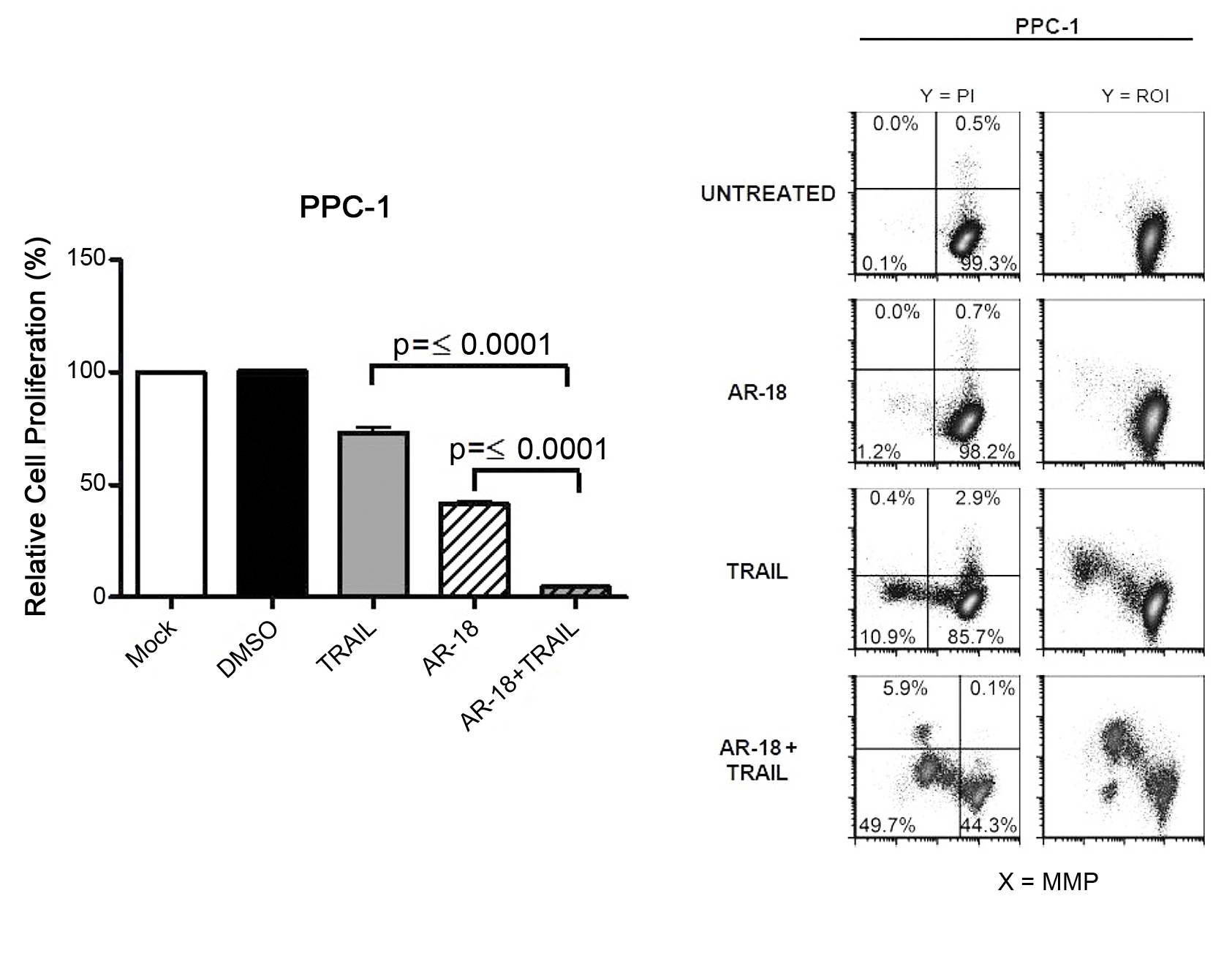

Supplement: Figure S1 — AR-18 sensitizes wild-type prostate cancer cell line PPC-1 to TRAIL. Left panel: PPC-1cells were treated with TRAIL for 24 h after a 24 h pre-exposure to AR-18 at the indicated concentrations, and cell viability measured by SRB assay. Each bar graph signifies mean from three separate experiments with six replicates. error bars = ± SEM. Right panel: PPC-1 cells were untreated or incubated with AR-18 (25 µM), TRAIL (10 ng/mL), or their combination for 24 h, and then analyzed by flow cytometry. TRAIL induced loss of ΔΨm and combined treatment with TRAIL and AR18 clearly sensitized the cell line to loss of ΔΨm, and this was accompanied by increased ROI generation and loss of out membrane integrity. (TIF) [file pone.0041102.s001.tif]

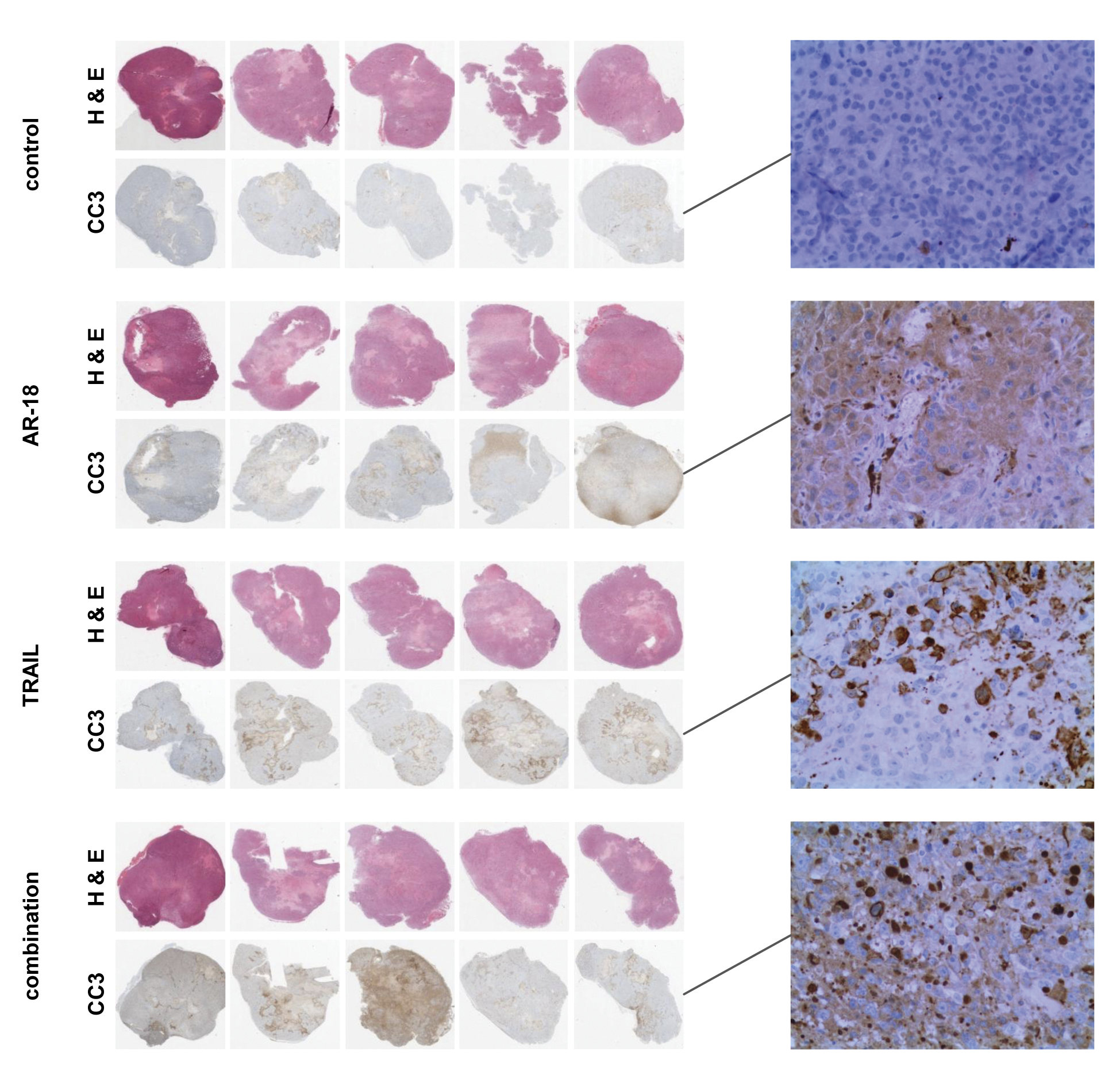

Supplement: Figure S2 — Synergistic interaction of GSK-3 and TRAIL in apoptosis induction in vivo . Immunohistochemistry staining of cleaved caspase-3 and H&E in PANC-1 s.c. tumor xenografts in male SCID mice. Groups of five mice were i.p. treated with acute doses of DMSO (control), AR-18, TRAIL or their combination, as depicted in Figure 6A. Tumors were excited, formalin fixed, stained and visualized under an Olympus BX41 microscope. A representative section imaged using a 40X magnification objective lens from one mouse in each group is presented to the right. (TIF) [file pone.0041102.s002.tif]

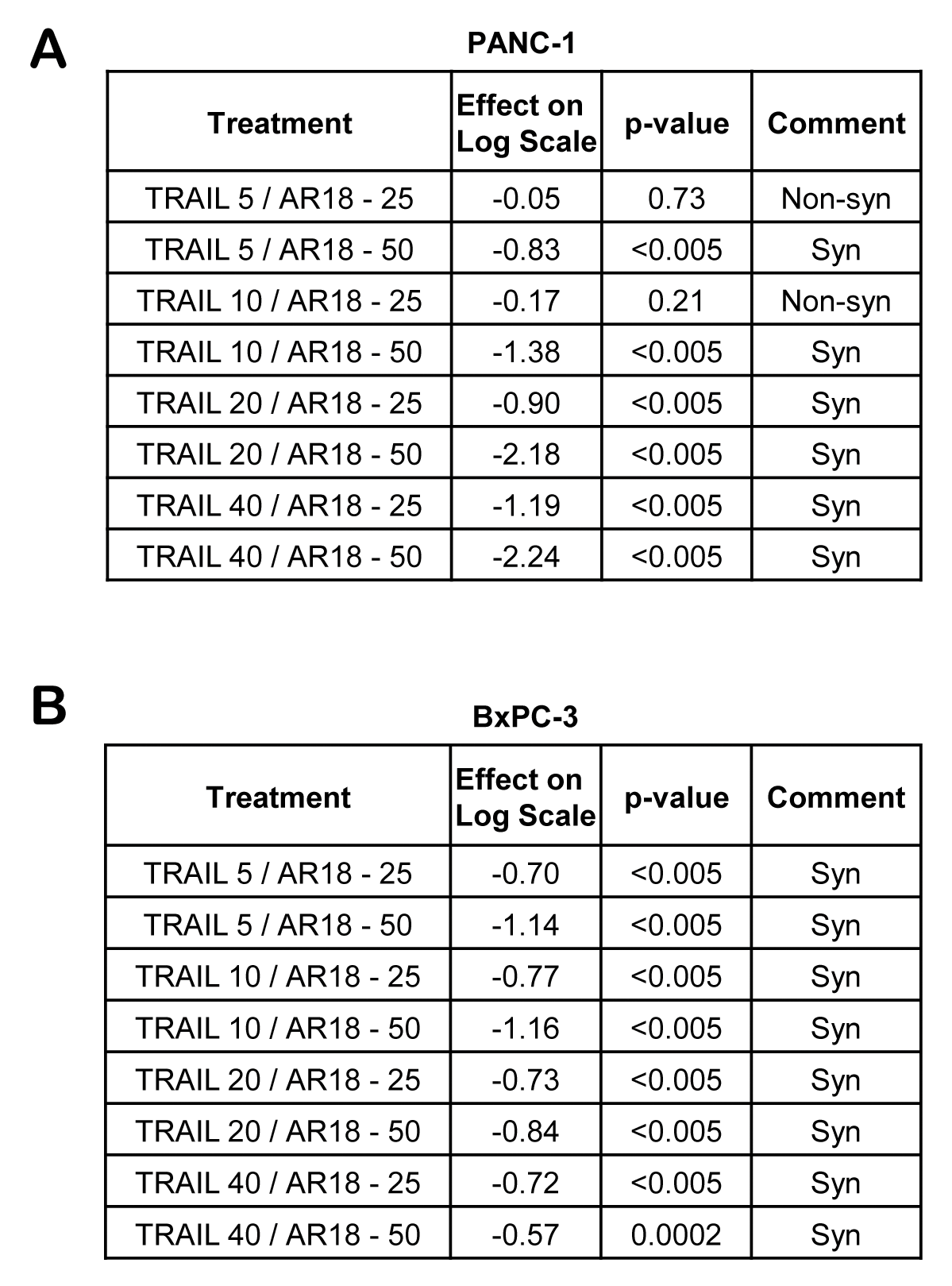

Supplement: Table S1 — Statistical analysis indicates synergistic interaction when AR-18 and TRAIL are combined. To determine the potential synergistic effect of the AR-18 and TRAIL combination, the SRB cell proliferation data from PANC-1 and BxPC-3 (Tables A and B respectively) were subjected to statistical analysis by log transforming the data and using linear regression model. Syn: synergistic effect, Non-syn: Not synergistic. (TIF) [file pone.0041102.s003.tif]
